# Mobile Clinical Decision Tools Among Emergency Department Clinicians: Web-Based Survey and Analytic Data for Evaluation of The Ottawa Rules App

**DOI:** 10.2196/15503

**Published:** 2020-01-29

**Authors:** Amanda My Linh Quan, Ian Stiell, Jeffrey J Perry, Michelle Paradis, Erica Brown, Jordan Gignac, Lindsay Wilson, Kumanan Wilson

**Affiliations:** 1 The Ottawa Hospital Research Institute Clinical Epidemiology Ottawa, ON Canada; 2 University of Ottawa Department of Emergency Medicine Ottawa, ON Canada

**Keywords:** emergency departments, mHealth, clinical prediction rule, decision aids

## Abstract

**Background:**

The Canadian CT Head Rule (CCHR), the Canadian Transient Ischemic Attack (TIA) Score, and the Subarachnoid Hemorrhage (SAH) Rule have all previously demonstrated the potential to significantly standardize care and improve the management of patients in emergency departments (EDs). On the basis of user feedback, we believe that the addition of these rules to the *Ottawa Rules App* has the potential to increase the app’s usability and user acceptability.

**Objective:**

This study aimed to evaluate the perceived usefulness, acceptability, and uptake of the enhanced *Ottawa Rules App* (which now includes CCHR, TIA, and SAH Rules) among ED clinicians (medical students, residents, nurses, and physicians).

**Methods:**

The enhanced *Ottawa Rules App* was publicly released for free on iOS and Android operating systems in November 2018. This study was conducted across 2 tertiary EDs in Ottawa, Canada. Posters, direct enrollment, snowball sampling, and emails were used for study recruitment. A 24-question Web-based survey was administered to participants via email, and this was used to determine user acceptability of the app and Technology Readiness Index (TRI) scores. In-app user analytics were collected to track user behavior, such as the number of app sessions, length of app sessions, frequency of rule use, and the date app was first opened.

**Results:**

A total of 77 ED clinicians completed the study, including 34 nurses, 12 residents, 14 physicians, and 17 medical students completing ED rotations. The median TRI score for this group was 3.38, indicating a higher than average propensity to embrace and adopt new technologies to accomplish goals in their work or daily lives. The majority of respondents agreed or strongly agreed that the app helped participants accurately carry out the clinical rules (56/77, 73%) and that they would recommend this app to their colleagues (64/77, 83%). Feedback from study participants suggested further expansion of the app—more clinical decision rules (CDRs) and different versions of the app tailored to the clinician role. Analysis and comparison of Google Analytics data and in-app data revealed similar usage behavior among study-enrolled users and all app users globally.

**Conclusions:**

This study provides evidence that using the *Ottawa Rules App* (version 3.0.2) to improve and guide patient care would be feasible and widely accepted. The ability to verify self-reported user data (via a Web-based survey) against server analytics data is a notable strength of this study. Participants’ continued app use and request for the addition of more CDRs warrant the further development of this app and call for additional studies to evaluate its feasibility and usability in different settings as well as assessment of clinical impact.

## Introduction

### Background

Clinical decision rules (CDRs) are useful tools in emergency departments (EDs) as they can objectively guide clinicians in making critical decisions regarding patient care. There is evidence that the appropriate application of CDRs can aid in standardization of practice and improve patient care, leading to a reduction in ED wait times and significant health cost savings [1-3]. These findings, combined with the growing ubiquity of mobile phone technology in health care settings [4], have provided clinicians with a means of improving patient care by rapidly and easily accessing CDRs and reducing health care costs. Our previous work to make CDRs more accessible by developing the *Ottawa Rules App* was evaluated in phase I of the *Ottawa Rules Study* [5]*.* Phase I work was centered around development and evaluation of the *Ottawa Rules App (version 1.0.0),* which housed 3 validated ED clinical rules—The Ottawa Knee Rule [6], Ottawa Ankle Rules [7], and Canadian C-Spine Rule [8], collectively known as The Ottawa Rules. *The Ottawa Rules App (version 1.0.0)* was well received; it was found to be helpful in applying the rules, and the large majority of participants would recommend the app to colleagues.

### Objectives

Phase I user feedback indicated that that the addition of The Canadian CT Head Rule (CCHR) [9], the new Canadian Transient Ischemic Attack (TIA) Score, and Subarachnoid Hemorrhage (SAH) Rule [10] to the app had the potential to increase the app’s usability and user acceptability*.* These rules have previously demonstrated the potential to significantly standardize care and improve the management of patients in EDs [9] and thus further reduce unnecessary radiographic imaging at The Ottawa Hospital (TOH) and beyond. In this study, we sought to develop and add the CCHR, TIA Score, and SAH rules for use in the app, as well as evaluate the enhanced app.

*The Ottawa Rules Study: Phase II* aims to pilot *The Ottawa Rules App* (Version 3.0.2) among ED clinicians (physicians, residents, nurses, and medical students) at TOH in Ottawa, Canada, and evaluate the perceived usefulness, acceptability, and uptake of the enhanced *Ottawa Rules App* (version 3.0.2).

## Methods

### Mobile App Improvements

Primary development of the CCHR, TIA Score, and SAH rules for use in the* Ottawa Rules App* (Version 3.0.2) was completed in the middle of 2018. The enhanced app underwent internal user testing and iteration among a small group of ED physicians before the app’s public release. The app’s mechanism for feedback and user support, where users were permitted to provide suggestions for app improvements, to report bugs, and to request for technical assistance remained unchanged from phase I [5]. The final enhanced version of the* Ottawa Rules App* (version 3.0.2)*,* which was used for this study, added CCHR, TIA Score, SAH rules, and new user interactive features and updated Nursing Directives. This version was completed in November 2018.

### App Release and Promotion

The enhanced *Ottawa Rules App* (version 3.0.2) was publicly released for iOS devices via the App Store, for Android devices via Google Play, through TOH Research Institute (OHRI) app portal, and on the Ottawa Rules website [1]. On the basis of the success of the phase I app release and its associated promotional activities, the enhanced *Ottawa Rules App* was promoted through institutional, local, and national channels. Institutional emails were circulated to all ED medical students, residents, nurses, and physicians, and the app’s public release was featured in the weekly TOH news release, “What’s Happening.” Additional promotional efforts were made across social media channels, including Twitter and Facebook.

### Study Enrollment

During the enrollment period (November 15, 2018-May 15, 2019)*,* various recruitment strategies were used to enroll participants from the 2 campuses (Civic and General) that comprise TOH. These strategies included posters, direct enrollment (study coordinators approaching eligible ED clinicians during work hours), snowball sampling, and emails. Study inclusion criteria were as follows: above 18 years of age, working as a clinician (medical student, resident, nurse, or physician) or be on rotation in a TOH ED, possess an institutional email (TOH, OHRI, or University of Ottawa email address), and own a personal or institutional iOS or Android smartphone onto which they could download the enhanced *Ottawa Rules App* (version 3.0.2).

**Figure 1:**
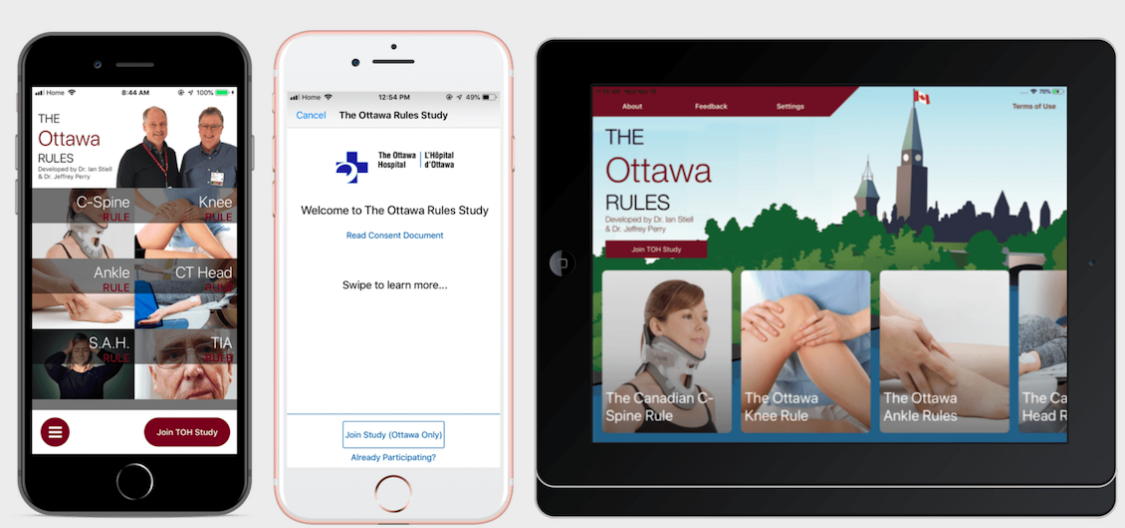
The *Ottawa Rules App* (Version 3.0.2) interfaces on iOS. The Ottawa Rules is available as a free download for mobile and tablet devices. A ‘TOH Study’ button was available on the app homepage for interested participants who were then prompted to review an in-app consent document before enrolment.

Individuals’ study eligibility was assessed as part of the in-app informed consent process. Figure 1 provides screenshots of the *Ottawa Rules App* (version 3.0.2) landing page where the “TOH Study” button would then consecutively lead participants through study information screens and study enrollment and consent forms. Consenting participants then had to verify their institutional email by clicking a verification link sent to the institutional email provided, before being enrolled. Individuals who did not meet the study inclusion criteria were notified as such and were unable to move further through the enrollment process. Participants had in-app access to the consent documents and contact information of study staff throughout the duration of the study. The Ottawa Health Research Network Research Ethic Board (#20150405-01H) approved the study.

### Data Collection

The methodology used to collect in-app analytics and user evaluation of the app did not differ much from phase I of *The Ottawa Rules Study.* In brief, user analytics (ie, number of app sessions, frequency of rule use, and the date the app was first opened) were collected and encrypted instantaneously before being sent to a secure cloud server in Canada, administrated by TOH mobile health Lab at OHRI. Feasibility, perceived user acceptability, and usability of the app were evaluated 1-month postenrollment via a Web-based survey. The survey was sent to participants’ verified institutional email and comprised 3 multiple-choice, 20 5-point Likert scale, and 2 open-ended questions. The Web-based survey used for this study can be viewed in Multimedia Appendix 1. Participants who completed the 1-month poststudy survey received an electronic coffee gift card worth Can $10 (US $8), the same amount as phase I. To assess participants’ “propensity to embrace and use cutting-edge technologies for accomplishing goals in home, life and at work,” the Technology Readiness Index (TRI) 2.0 was administered in the second half of the Web-based survey (Questions 10-25) [11].

Google Analytics (GA) was also used to obtain app usage statistics and understand how users globally utilize the *Ottawa Rules App* (version 3.0.2). GA’s behavior flow feature also allowed us to track and visualize the path users traveled through the app—from the home page to the rules or other TOH resources. Having GA data (external data), in-app analytic data, and survey data (internal data) allowed for data triangulation [12]. This comparison was used to establish degree of compatibility and generalizability of results.

## Results

### Participants

**Figure 2:**
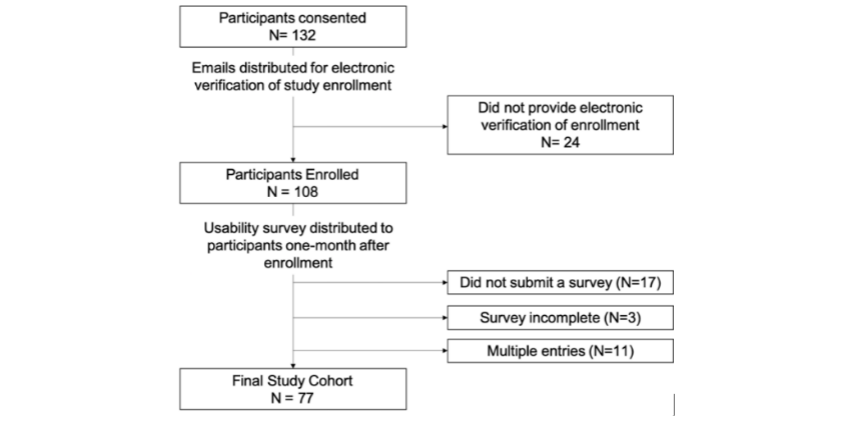
Study flow diagram.

A total of 132 participants from the 2 TOH campuses (Civic and General) met the eligibility criteria and provided electronic consent to join the study (Figure 2). Study participants were excluded from the final study cohort if they did not submit the 1-month poststudy survey or if their survey was incomplete. The final study cohort comprised 77 participants (Figure 1). Nurses constituted the largest proportion of study participants (34/77, 44%), followed by physicians (14/77, 18%), medical students (17/77, 22%), and residents (12/77, 16%). Participant characteristics are summarized in Table 1.

**Table 1:** Descriptive characteristics of survey respondents (N=77).

Characteristics		Respondents	
**Level of training, n (%)**			
	Medical student		17 (22)
	Nurse		34 (44)
	Physician		14 (18)
	Resident		12 (16)
**Age range (years), n (%)**			
	18-24		10 (13)
	25-34		41 (53)
	35-44		17 (22)
	45-54		7 (9)
	55-64		2 (3)
**Years of service, n (%)**			
	<1		12 (16)
	1-5		42 (55)
	6-10		11 (14)
	11-15		5 (7)
	16-20		2 (3)
	≥21		5 (7)
**Sex, n (%)**			
	Female		51 (66)
	Male		26 (35)

### Usability Survey

A total of 72% (56/77) of the participants agreed or strongly agreed that the app helped participants accurately carry out the clinical rules, and more than 75% (58/77) of the participants agreed or strongly agreed that they would recommend this app to their colleagues. In addition, 84% (65/77) of the participants agreed or strongly agreed that they would continue using the app. More than half, 55% (42/77), of the study participants reported that they used the app weekly, 34% (26/77) of the participants said they used the app monthly, 3% (2/77) of the participants said they used the app daily, and 9% (7/77) of the participants said they never used the app. Only 1% (1/77) users reported difficulty using the app. Although there was favorable reception of the app by ED clinicians, only 39% (30/77) of the participants agreed or strongly agreed that they used the app for the majority of the cases that required use of the clinical rules. The C-Spine rule was reported as the most useful rule by users. See Multimedia Appendix 1 for full survey results.

Participants provided numerous positive comments regarding the usability of the *Ottawa Rules App (*Version 3.0.2), and they generally had little difficulty using the app. A participant commented, “Just keep on expanding the app as more CDRs arise.” Participants liked the app and several of them suggested that the app should be built into “Epic,” TOH’s “1 patient-1 record” electronic health information system. Being able to quickly move through the rule inclusion and exclusion checklist and recommended clinical strategies was beneficial and described as “[This app is] perfect for triage nurses.” Participants also reported that the app could be beneficial for those who are still learning the rules. For example, a participant mentioned, “Great for teaching. Consider pushing the app out to learners” and another said, “…as a new medical student it was convenient and comforting to have that information at my fingertips.” Users also suggested that rule feedback should be modified, as they found it to be more physician focused; a nurse explained “…nursing cannot order a CT, we are most interested in whether the patient needs a collar or not. Using the app with a colleague the other day, once I reworded it for her as ‘can we say that the patient is unlikely to have an injury (no collar) or are they likely and will need a CT to make the diagnosis (collar)?.”

### Technology Readiness Index

As part of the survey, we also measured participants’ innate propensity to adopt and utilize a new technology to achieve a goal at home or in their work life by using the TRI 2.0 [11]. The mean TRI score among the final study cohort was 3.38 (IQR 0.69, SD 0.47) on a 1 to 5 scale. The mean overall TRI score of the US population was reported as 3.02 in 2014 [11]; thus, our study participants demonstrated relatively higher than average propensity to embrace/adopt new technologies to accomplish goals in their work or daily lives. A Pearson chi-square test revealed significant correlation (χ^2^_104_=12.4, *P*=.42) between TRI scores and participant age, with younger participants having higher scores.

### In-App Activity

Over 7 months, study-enrolled participants accessed the app 489 times. Most participants returned to the app multiple times over the 8-month period: 40% (31/77) of the participants used the app on between 2 and 4 days, and 14% (11/77) of the participants used the app on 5 or more days. Server data showed that some participants did not engage with any specific app features—15 participants (15/77, 19%) did not venture beyond the home screen, which is slightly higher than self-reported nonuse. The newly added rules (CCHR, TIA score and SAH rules) were collectively accessed a total of 197 times (40% of total app uses). Participants’ app engagement stratified by content accessed and clinician role is presented in Table 2. Nurses were the most active users; they accounted for 44% of all users and 37% of all app uses.

**Table 2:** Study user app engagement stratified by content accessed and clinician role.

Rules	All clinicians, n (%)	Students, n (%)	Residents, n (%)	Nurses, n (%)	Physicians, n (%)
All Rules Total	489 (100)	120 (24.5)	110 (22.5)	181 (37.0)	78 (16.0)
The Ottawa Rules Total	187 (100)	51 (27.3)	41 (21.9)	71 (38.0)	24 (12.8)
Ankle Rules	63 (33.7)	13 (20.6)	11 (17.4)	34 (53.9)	5 (7.9)
Knee Rule	42 (62.7)	17 (40.4)	7 (16.6)	12 (28.6)	6 (14.3)
C-Spine Rules	82 (43.9)	21 (25.6)	23 (28.0)	25 (30.5)	13 (15.6)
Newly added rules Total	197 (100)	51 (25.9)	51 (25.9)	52 (26.4)	43 (21.8)
Canadian CT Head Rule	89 (45.2)	23 (25.8)	27 (30.3)	18 (20.2)	21 (23.6)
Transient Ischemic Attack	48 (24.4)	9 (18.8)	6 (12.5)	15 (31.3)	18 (37.5)
Subarachnoid Hemorrhage Rule	60 (30.5	19 (31.7)	18 (30.0)	19 (31.7)	4 (6.7)
Other TOH resources^a^	105 (100)	18 (17.1)	18 (17.1)	58 (55.2)	11 (10.5)

^a^Refers to the Ottawa Hospital (TOH) nursing directives, antibiotic guidelines, and triage algorithms.

### Triangulation With Google Analytics Data (Global App Use)

**Figure 3:**
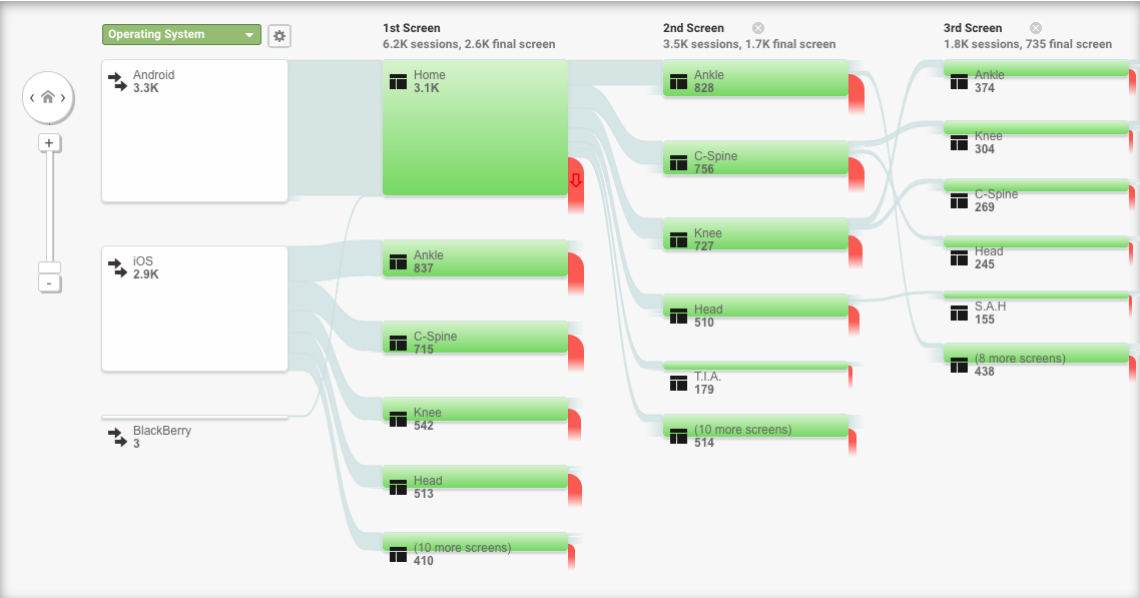
Behaviour flow diagram stratified by user operating system.

GA was used to obtain app usage statistics and understand how all users utilized the *Ottawa Rules App* (Version 3.0.2) during the study period (Figure 3). Aggregated app usage data among all users between November 15, 2018, and May 1, 2019, were retrieved. During this time, 48,349 app sessions were recorded among 42,225 app users. A large majority of users, 94.5% (40,096/42,225), are based in the United States. The app was visited by a minimum of 44 users and a maximum of 669 users per day, with a minimum of 45 sessions and a maximum of 695 sessions per day. The average app session length was 59 seconds.

In-app data revealed that among study-enrolled users, CCHR, C-Spine, SAH Rule, and Ankle rules (in order of most use) were the most frequently accessed rules on the app (Table 2). GA data revealed similar usage trends among users globally—the Ankle, C-Spine, Knee, and CCHR rules were the most frequently used rules.

## Discussion

### Principal Findings

This study generated evidence on acceptability and feasibility of operationalization of a CDR app, the* Ottawa Rules App* (version 3.0.2), among ED clinicians to guide patient care. Quantitative and qualitative findings suggest that participants believed the app facilitated accurate use of the CDRs. Whether participants used the app for the majority of their cases where the CDRs were applicable is unclear. Overall, survey data suggest that the app was useful in guiding clinical decision making, and it is a tool that clinicians would use in the future and would recommend to colleagues. These findings are consistent with the positive reception and usage patterns reported in phase I [5]. Analysis and comparison of GA data and in-app data revealed similar usage behavior among study-enrolled users and all app users globally. The addition of the CCHR, TIA, and SAH rules to the app proved to be beneficial, as these rules were collectively accessed just as much as the Ottawa Rules (Table 2).

The wide adoption of smartphones by health care professionals for use in medical practice has been widely documented in recent years [13]. Implementation studies have demonstrated that the use of CDRs in the ED can result in a relative reduction of radiography for ankle, knee, and cervical spine injuries [14] by 26.4%, 26%, and 15.5%, respectively [15,16]. Uptake and implementation of CDRs has been less optimal, but mobile apps provide a unique opportunity to increase and improve CDR use by facilitating access. This study demonstrated feasibility and acceptability of using a mobile app to access and use 6 validated CDRs. Our findings are consistent with previous studies that have shown that smartphone-based apps are an acceptable and effective modality for quickly and easily accessing electronic resources to support and guide patient care [17,18]. The ability to verify self-reported user data (via Web-based survey) against server analytics data is a notable strength of this study. In addition, global app usage as monitored by GA demonstrates some generalizability. Our methodology and findings add to a growing body of literature on how evaluation and validation of smartphone apps for medical care provider use can be done [19,20].

### Limitations

This study has some limitations. First, as with many studies of this nature, limitations related to generalizability exist. The use of GA data to demonstrate similar app usage trends compared with our analysis and in-app data partially addresses this issue. Further work comparing the usage data between the hospital users and the global users could further address the issue of generalizability; however, hospital users only make up about 0.5% of all users worldwide. Second, response bias, as well as familiarity bias, may exist, as all the rules housed in the piloted version of the app were developed by clinicians at TOH, and clinicians who participated in this study may have also participated in phase I piloting. To overcome these biases, future work to evaluate feasibility and acceptability of the *Ottawa Rules App* (version 3.0.2) should be done externally among clinicians who are less likely to be familiar with the CDRs housed in the app. Given the well-documented impact of CDR use and potential for cost savings, improving CDR accessibility is important. Mobile technology offers the opportunity to facilitate access and use of CDRs; this study demonstrates that this is feasible. Future research should weigh the potential advantages of integrating CDRs into electronic medical record (EMR) systems and adding more CDRs to the app. A formal study evaluating the impact of CDR use, facilitated by a mobile app, on clinical care is needed to establish validity. In addition, more evidence is needed to support whether CDR use in EDs, facilitated by mobile apps, will translate into real reductions in unnecessary diagnostic imaging and health care costs. Future work should explore the use of the app in other settings and its impact on health services utilization and patient outcomes from a system or macro-level perspective. Integration of the digital tool into EMRs may also facilitate use and would be worth evaluating.

This study provides evidence that the use of *Ottawa Rules App* (version 3.0.2) to improve and guide patient care would be feasible and widely accepted. The addition of CCHR, TIA score and SAH rules for use in the app had favorable reception. Participants’ continued app use (as reported by the Web-based survey) and demand for the addition of more CDRs warrant the further development of this app and call for additional studies to evaluate its feasibility and usability in different settings. Uptake and implementation of CDRs has not been optimal; this app offers a way to make use of mobile apps to facilitate use of CDRs to standardize patient care in EDs and reduce unnecessary radiographic imaging.

## Appendix

Multimedia Appendix 1 One-month post enrolment usability survey questions and response data.

## Abbreviations

CCHR: Canadian CT Head Rule
CDR: clinical decision rule
ED: emergency department
EMR: electronic medical record
GA: Google Analytics
OHRI: Ottawa Hospital Research Institute
SAH: Subarachnoid Hemorrhage
TIA: Transient Ischemic Attack
TOH: The Ottawa Hospital
TRI: Technology Readiness Index
