# Supplementary material for: Mobile Clinical Decision Tools Among Emergency Department Clinicians: Web-Based Survey and Analytic Data for Evaluation of The Ottawa Rules App
Source: JMIR Mhealth Uhealth. 2020 Jan 29;8(1):e15503. doi: 10.2196/15503 (PMC7016628; doi:10.2196/15503)
Supplement: Multimedia Appendix 1 [file mhealth_v8i1e15503_app1.docx]

The Ottawa Rules PHASE II Survey Questions and Results

Q1. I used the app for the majority of the cases that required these clinical rules.

Q2. The app was useful in helping me accurately carry out these clinical rules.

Q3. I would recommend this app to my fellow colleagues.

Q4. I will continue using this app.

Q5. Which rules did you find the most useful?

Q6. Approximately how frequently did you use the app?

Q7. Did you have any issues using the app?

Q8, Q9: open ended questions

TRI portion of survey

Q10. New technologies contribute to a better quality of life.

Q11. Technology gives me more freedom of mobility.

Q12. Technology gives people more control over their daily lives

Q13. Technology makes me more productive in my personal life

Q14. Other people come to me for advice on new technologies.

Q15. In general, I am among the first in my circle of friends to acquire new technology when it appears.

Q16. I can usually figure out new high-tech products and services without help from others.

Q17. I keep up with the latest technological developments in my areas of interest

Q18. When I get technical support from a provider of a high-tech product or service, I sometimes feel as if I am being taken advantage of by someone who knows more than I do.

Q19. Technical support lines are not helpful because they don't explain things in terms I understand.

Q20. Sometimes, I think that technology systems are not designed for use by ordinary people.

Q21. There is no such thing as a manual for a high-tech product or service that's written in plain language.

Q22. People are too dependent on technology to do things for them.

Q23. Too much technology distracts people to a point that is harmful.

Q24. Technology lowers the quality of relationships by reducing personal interaction.

Q25. I do not feel confident doing business with a place that can only be reached online.
